# Deciphering Hydrogen Embrittlement Mechanisms in Ti6Al4V Alloy: Role of Solute Hydrogen and Hydride Phase

**DOI:** 10.3390/ma17051178

**Published:** 2024-03-03

**Authors:** Tien-Dung Nguyen, Chetan Singh, Dong-Hyun Lee, You Sub Kim, Taeho Lee, Soo Yeol Lee

**Affiliations:** Department of Materials Science and Engineering, Chungnam National University, Daejeon 34134, Republic of Korea; dungnt.mse@gmail.com (T.-D.N.); csingh@mt.iitr.ac.in (C.S.); dhlee@cnu.ac.kr (D.-H.L.); usube2012@o.cnu.ac.kr (Y.S.K.); dlxogh1021@o.cnu.ac.kr (T.L.)

**Keywords:** Ti6Al4V, hydrogen embrittlement, solute hydrogen, hydride phase

## Abstract

Ti6Al4V (Ti64) is a versatile material, finding applications in a wide range of industries due to its unique properties. However, hydrogen embrittlement (HE) poses a challenge in hydrogen-rich environments, leading to a notable reduction in strength and ductility. This study investigates the complex interplay of solute hydrogen (SH) and hydride phase (HP) formation in Ti64 by employing two different current densities during the charging process. Nanoindentation measurements reveal distinct micro-mechanical behavior in base metal, SH, and HP, providing crucial insights into HE mechanisms affecting macro-mechanical behavior. The fractography and microstructural analysis elucidate the role of SH and HP in hydrogen-assisted cracking behaviors. The presence of SH heightens intergranular cracking tendencies. In contrast, the increased volume of HP provides sites for crack initiation and propagation, resulting in a two-layer brittle fracture pattern. The current study contributes to a comprehensive understanding of HE in Ti6Al4V, essential for developing hydrogen-resistant materials.

## 1. Introduction

Ti6Al4V (Ti64) is a highly relevant material that is lauded for its unique properties in a variety of fields, including aerospace, manufacturing, and the biomaterial industry [1,2,3]. The coexistence of the hexagonal close-packed phase (α) and body-centered cubic phase (β) results in excellent specific strength and superior corrosion resistance [4]. Therefore, Ti64 is a strong contender for sustainable development in the aerospace sector by reducing fuel consumption. The proven resilience in demanding conditions and adherence to stringent mechanical specifications make Ti64 highly suitable for various applications within the aerospace sector [1]. However, hydrogen embrittlement (HE) remains a significant challenge that adversely affects the performance of the components working in hydrogen environments [5,6,7,8,9]. Exceeding a critical hydrogen concentration triggers hydrogen embrittlement in Ti64, causing a substantial reduction in both strength and ductility.

It has been reported that the behavior of hydrogen embrittlement is directly influenced by the interaction of hydrogen with both initial and deformed microstructures. Previous studies have predominantly focused on elucidating the interplay between hydrogen and microstructural features [7,10,11,12,13,14]. In titanium alloys, the difference in the diffusion rate and solubility of hydrogen between the hexagonally close-packed α and body-centered cubic β phases lead to a notable discrepancy in the concentration of hydrogen within these two phases. The β phase has been recognized as a reservoir for hydrogen [11], while specific orientation relationships have been identified for preferential hydride precipitation within the α phase [7,12]. Moreover, HE is strongly affected by the presence of either solute hydrogen (SH) or hydride phase (HP), depending on the amount of hydrogen permeating the material. Specifically, SH can induce crystal defects and nano- or micro-voids [10,13]. Upon surpassing a certain threshold, HP formation leads to volume expansion and lattice strains, as reported by Deconinck et al. [7]. As a consequence, the formation of numerous nano- or micro-cracks within the HP layer enables the accommodation of stresses [7]. However, the existing literature provides limited insights into the impact of SH and HP on the mechanical properties. Particularly, there seems to be a noticeable absence of comprehensive studies delving into a comparative analysis of their distinct influences on HE behaviors. Previous studies have mainly concentrated on the effects of hydrogen charging on the interaction of hydrogen with microstructure, leading to the formation of HP or the mere presence of SH [5,7,10,13,15]. Some studies have also indicated the roles of HP in reducing mechanical properties through tensile tests [6,8,16,17]. Exploring the formation of different hydrogen variants and their mechanisms influencing HE behavior has remained elusive. Understanding these variations offers numerous advantages for predicting the intricate performance and lifespan of the components in the diverse hydrogen environments.

In this study, we meticulously examined the effects of different presences of hydrogen on the deformation behavior of Ti64 alloy at room temperature. Both SH and HP were induced through a precise control via electrochemical hydrogen charging. Subsequently, we assessed micro- and macro-mechanical properties using nanoindentation measurements and uniaxial tensile tests on both hydrogen-charged and uncharged samples. The current findings demonstrate that hydrogen-charged samples exhibit discernible brittle fracture characteristic differences between HP and SH.

## 2. Materials and Methods

A Ti64 (ASTM B265) plate (300 × 100 × 10(t) mm^3^) was produced by rolling at 950 °C for 2.5 h, followed by annealing at 750 °C for 2 h. Electric discharge machining was used to fabricate plate-type dog-bone-shaped tensile samples. The dimension of the gauge section was 12.5 × 6 × 1(t) mm^3^, in which the representative surface was on the rolling-direction–transverse-direction plane (RD–TD plane). Hydrogen induction was accomplished through the electrochemical hydrogen-charging method. The electrolyte contains 0.5 mol/L H_2_SO_4_ and 3 g/L NH_4_SCN (thiourea). Thiourea was intentionally introduced to amplify the uptake of atomic hydrogen into the materials, exploiting its inhibitory action against the hydrogen recombination process that results in the creation of molecular H_2_ [7,18]. The galvanostatic mode was employed at two different current densities, specifically set at 1 and 50 mA/cm^2^ to introduce SH and HP, respectively. Moreover, the hydrogen-charging procedure persisted for 24 h at room temperature.

Nanoindentation tests (KLA Instruments, Milpitas, CA, USA) were conducted to examine micro-mechanical properties among the base metal, SH, and HP. All the measurements were performed at RT using a Berkovich diamond indenter with an indentation strain rate of 0.5/s, a maximum load of 5 mN, and a hold time of 1 s. Furthermore, the tensile tests were performed using a universal testing machine (UTM) (UT-O2OE) at RT, with a strain rate of 10^−5^/s, to examine the macro-mechanical behavior of both hydrogen-charged and uncharged samples. The selected slow strain rate was aimed at describing the influence of SH and HP on the HE.

X-ray diffraction (XRD) measurements were performed utilizing a Rigaku instrument (Tokyo, Japan). Cu Kα radiation was applied to scan the 2θ angle over the range of 30° to 80° with a step size of 0.01°. This procedure was aimed at identifying the phases present in both hydrogen-charged and uncharged samples. For microstructural examinations, the samples underwent mechanical polishing of up to 3 µm diamond suspension, followed by a final polishing step using a solution comprising 0.04 µm colloidal silica suspension of 50 mL, H_2_O_2_ (30%) of 10 mL, and Kroll’s reagent of 5 mL. To ascertain the presence of HP, microstructural characterization was performed separately for the uncharged and hydrogen-charged samples at the high current density of 50 mA/cm^2^, using a scanning electron microscope (SEM; Merlin compact, Carl Zeiss, Jena, Germany) equipped with electron channeling contrast imaging (ECCI) and electron backscatter diffraction (EBSD). EBSD analysis was conducted at a tilt angle of 70°. The post-processing phase was executed using the TSL OIM 7 data analysis software. To evaluate the distinct brittle features caused by SH and HP at micro- and macro-levels, indents and fractography observations were performed on both hydrogen-charged and uncharged samples, respectively. Finally, the hydrogen-assisted cracking behaviors were investigated by analyzing the propagation behaviour of secondary cracks observed from the cross-section of the hydrogen-charged samples.

## 3. Results and Discussion

### 3.1. The Effect of Current Density on the Formation of Solute Hydrogen and Hydride Phase

**Figure 1:**
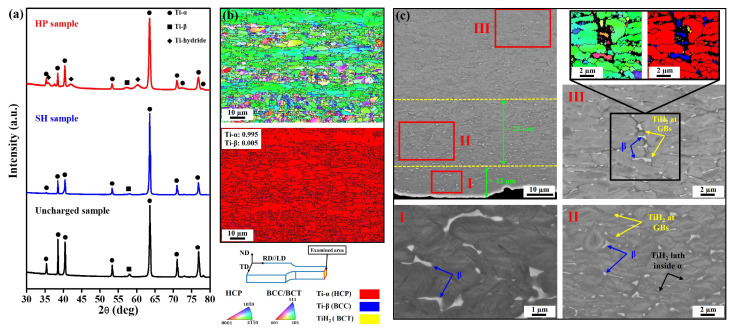
Microstructural analysis of the uncharged and the hydrogen-charged samples at the current density of 1 mA/cm^2^ (SH sample) and 50 mA/cm^2^ (HP sample) on the RD–TD plane: (**a**) XRD patterns of both hydrogen-charged and uncharged samples; (**b**) IPF and phase maps of the uncharged sample; (**c**) ECCI images and IPF and phase maps of the HP sample. Zone I: the HP fully transformed from α phase. Zone II: the HPs within the H-supersaturated α phase and at grain boundaries (GBs). Zone III: the HP at GBs.

Figure 1 illustrates XRD analysis and microstructural characterizations on the RD–TD plane of the uncharged sample and the samples charged at low current density of 1 mA/cm^2^ (SH sample) and high current density of 50 mA/cm^2^ (HP sample). The XRD patterns reveal that both hydrogen-charged and uncharged samples contain a majority of α phase with a small amount of β phase; however, small peaks of the HP (TiH_2_) are only observed in the HP sample (Figure 1a). The TiH_2_ has been demonstrated to be inducible through hydrogen charging and remains stable at RT [18,19,20,21,22,23]. Notably, the TiH_2_ exhibits a body-centered tetragonal (BCT) crystal structure with lattice parameters of a = 0.312 nm and c = 0.418 nm (JCPDS-PDF 9-371) [24]. This suggests that the HP is exclusively formed when charged with the high current density and the amount of HP fraction is gradually reduced as the measurement location moves away from the sample surface. In the sample charged at the low current density of 1 mA/cm^2^, the amount of hydrogen was insufficient to form the HP, indicating that only SH existed. The inverse pole figure (IPF) and phase map of the uncharged sample on the ND–TD plane show a random distribution of a minor β phase which is elongated along the α phase grains matrix (Figure 1b).

Due to the limited thickness of the HP layer and the hydrogen concentration being only near the hydrogen-charged surface, the evolution of HP formation was investigated by considering the cross-sectional area of the HP sample. Figure 1c shows the formation of HP that has three distinct zones in the HP sample. The morphological transformation of HP corresponds to changes in its fraction, depending on the distance from the sample surface. Hydrogen is unable to penetrate the center of the sample due to the slow diffusion rate of hydrogen in titanium at room temperature. The reported diffusion rates range from 3.26 × 10^−17^ m^2^/s to 1.05 × 10^−15^ m^2^/s in the α phase and from 2.17 × 10^−12^ m^2^/s to 2.32 × 10^−11^ m^2^/s in the β phase [11,25]. A similar evolution of HP formation during the hydrogen-charging process has also been documented by Deconinck et al. [7]. According to the ECCI and EBSD maps taken in zone III, the HP with a body-centered tetragonal lattice [22,23] develops at the interface of α/β phase or along grain boundaries (GBs). The β phase has much higher hydrogen diffusivity than the α phase and, therefore, hydrogen can easily accumulate at the α phase side near the α/β interface, exceeding its threshold value of solubility [7,11,18,25]. Moreover, GBs are reported as optimal pathways for accelerating the diffusion and accumulation of hydrogen [26,27,28,29]. As a result, the precipitation of HP primarily occurs either at the α–β interface or GBs. The presence of a zero solution (observed in the EBSD maps in Figure 1c) around the HP may be attributed to local strains within the HP, associated with volume expansion from the α phase-to-HP transformation [19,30]. Indeed, this transformation is reported to result in approximately an 18% volume expansion [11]. The volume effects lead to local plastic deformation of the matrix accompanied by an increase in dislocation density. With an increase in hydrogen concentration, HP preferentially forms in the α phase over the β phase. This is attributed to the smaller hydrogen solubility of the α phase (2 × 10^3^ wppm) compared to that of the β phase (20.6 × 10^3^ wppm) [7]. Zone II, characterized by a higher hydrogen concentration, leads to the development of HP within H-supersaturated α grains and at grain boundaries. Transitioning to Zone I, an abundant amount of hydrogen triggers the transformation of all α phase into HP. Notably, the β phase remains intact during the formation of HP (Figure 1c), indicating that the hydrogen concentration did not surpass the hydrogen solubility of the β phase.

### 3.2. Comparison of Nano-Mechanical Behavior by Nanoindentation

**Figure 2:**
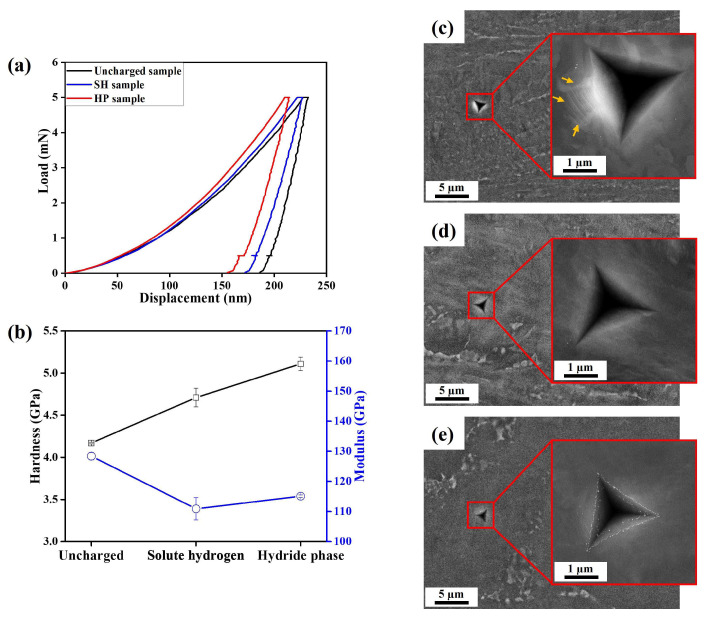
Nanoindentation measurement results: (**a**) load-displacement curves; (**b**) hardness and Young’s modulus; SEM images of indents in (**c**) the uncharged sample; (**d**) the SH sample; and (**e**) the HP sample. The orange arrows indicate pile-up.

Representative results of nanoindentation measurements are shown in Figure 2. The load-displacement curves highlight distinct micro-mechanical behaviors: the SH sample exhibited a slight decrease in maximum displacement, compared to the uncharged sample, whereas a significant drop was observed in the case of the HP sample (Figure 2a). This translates to the order of hardness values, shown in Figure 2b. It is reported that the HP exhibited higher inherent hardness compared to the base material [31]. On the other hand, it is known that the presence of SH leads to an increase in both dislocation density and dislocation mobility [32,33,34], which may contribute to a slight increase in the hardness of the SH sample. In comparison to the uncharged sample, the hydrogen-charged samples exhibited lower modulus values. The difference between SH and HP was within error margins. Within the hydrogen-affected layers, crack initiation and propagation may occur more easily than in the base metal, consequently leading to reduced resistance against elastic (recoverable) deformation under the indentation load [31]. Additionally, the indents of the hydrogen-charged samples consistently exhibit sink-in features, whereas the uncharged sample displays pile-up features along one edge of the indent (Figure 2c–e). Overall, the uncharged samples show an enhanced ductile plastic flow resulting in pile-up.

### 3.3. Comparison of Micro-Mechanical Behavior by Tensile Test

**Figure 3:**
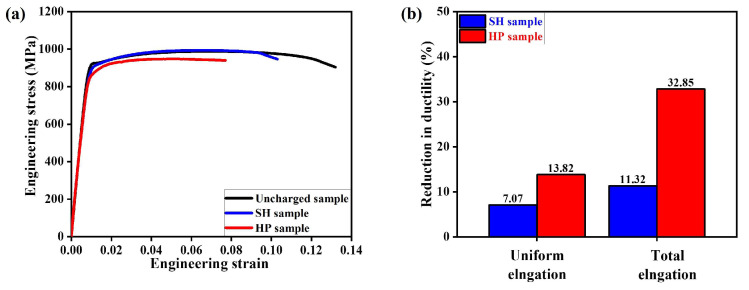
Comparison of mechanical properties among the uncharged sample, the SH sample, and the HP sample: (**a**) engineering stress–strain response; and (**b**) reduction in ductility from the stress–strain curve.

Figure 3 illustrates the mechanical properties of both hydrogen-charged and uncharged samples and Table 1 provides a summary of mechanical properties. The ratio (X_0_ − X_H_)/X_0_ (where X_0_ and X_H_ represent the elongation values for uncharged and charged samples, respectively) is calculated to estimate the reduction in ductility, as indicated in Figure 3b. In the HP sample, the strength slightly decreased compared to uncharged sample (uncharged sample: UTS = 986 ± 15 MPa and YS = 904 ± 16 MPa; HP sample: UTS = 963 ± 18 MPa and YS = 874 ± 10 MPa); however, the strength of the SH sample was very similar to the uncharged sample (SH sample: UTS = 1000 ± 6 MPa and YS: 900 ± 13 MPa). Due to the presence of hydrogen, both hydrogen-charged samples manifested a significant reduction in ductility. Specifically, the total elongation decreased by 13.82% in the SH sample and 32.85% in the HP sample, while the uniform elongation decreased by 7.07% and 11.32%, respectively, suggesting that HE induced by the HP is a more severe HE effect than that caused by the SH.

### 3.4. Fractography

**Figure 4:**
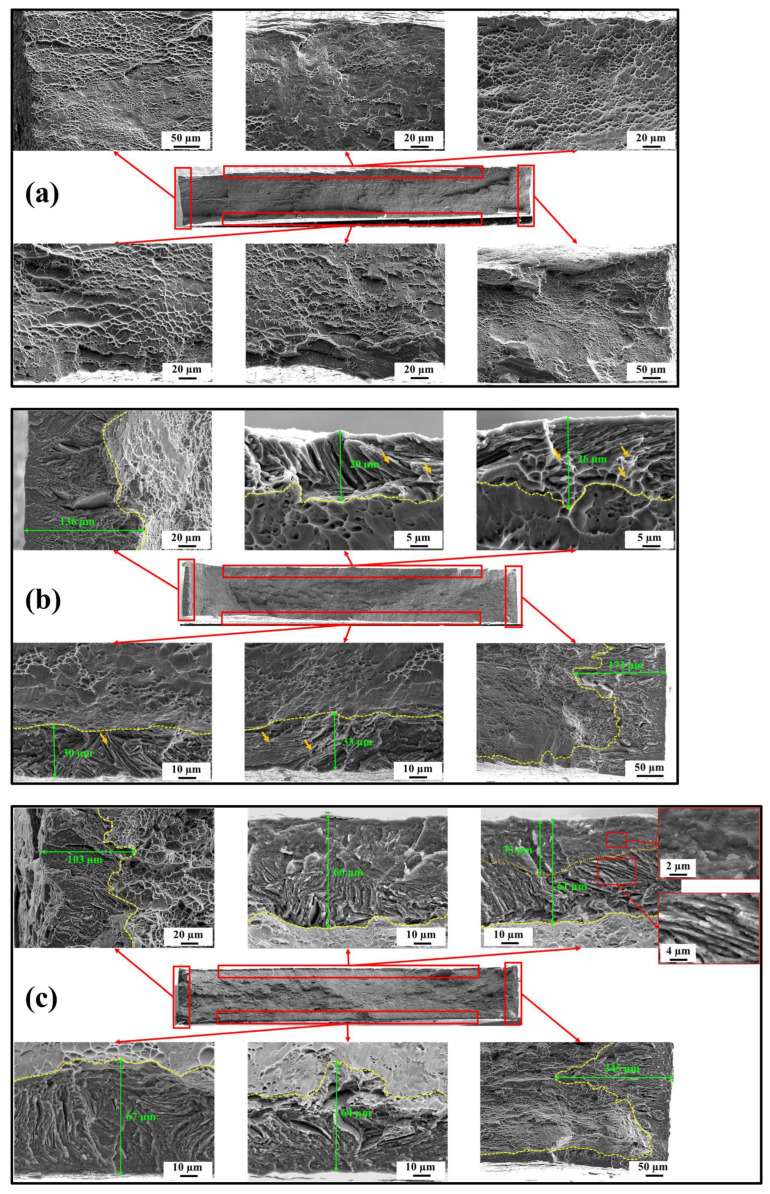
Fractography of (**a**) the uncharged sample; (**b**) the SH sample; and (**c**) the HP sample. The orange arrows indicate nano/micro-voids.

The distinct effects of SH and HP on microstructural deformation are elucidated by examining fractured surfaces of both hydrogen-charged and uncharged samples, as shown in Figure 4. The uncharged sample displays completely ductile behavior, characterized by the presence of dimples throughout the fractography (Figure 4a). In contrast, the hydrogen-charged samples exhibited partially brittle fracture morphology near the surface and the edges of the tensile samples (Figure 4b,c). In the fractography of the SH sample, colony patterns are discernible in the brittle layer (Figure 4b), resembling deformed microstructure of the uncharged sample (as described in the subsequent section). Furthermore, the presence of nano/micro-voids and micro-cracks at the grain boundaries provides an evidence of the influence of SH on HE. From previous studies, two perspectives have been reported regarding the impact of SH. Brosh et al. reported that SH forms by the hydrogen-charging process [13]. Under loading conditions, these nano/micro-voids may contribute to promoting cracking propagation. On the other hand, Ding et al. reported that SH enhances grain boundary vacancies during deformation, leading to a transition from transgranular to intergranular fracture [35]. Under applied load, these nano/micro-voids can function as structural vulnerabilities where cracks easily propagate or serve as possible sites for the initiation of micro-cracks. Overall, whether examining the influence of SH from either of the two perspectives, both acknowledge the impact of SH in supporting intergranular hydrogen-assisted cracking. However, the HP sample exhibits a two-layer brittle fracture pattern (Figure 4c). In the initial layer, cleavage is the predominant fracture mode, while intergranular fracture is evident in the layer below. Notably, the first layer corresponds to the combined zones I and II of the HP sample (Figure 1c), where local strains within HP can facilitate the nano/micro-cracks. During deformation, these cracks can play a role in affecting both crack initiation and propagation. The second layer belongs to zone III of the HP sample, where the HP at GBs can serve as pathways for crack propagation. As a result, both crack initiation and crack propagation in the HP sample occur much faster than those in the SH sample.

Additionally, the hydrogen-damaged region was examined in both hydrogen-charged samples. Owing to the varied distribution of hydrogen, distinct hydrogen-affected layers with varying depths were observed. In the SH sample, the thickness of the brittle layer ranged from 20 to 33 µm near the surfaces and from 136 to 172 µm near the edges. Meanwhile, in the HP sample, the brittle layer exhibited wider dimensions, ranging from 61 to 67 µm near the surface and from 103 to 345 µm near the edge, respectively. It can be concluded that the observed brittle features of the HP confirm its heightened susceptibility to HE compared to the SH.

### 3.5. Hydrogen-Assisted Cracking Behavior

**Figure 5:**
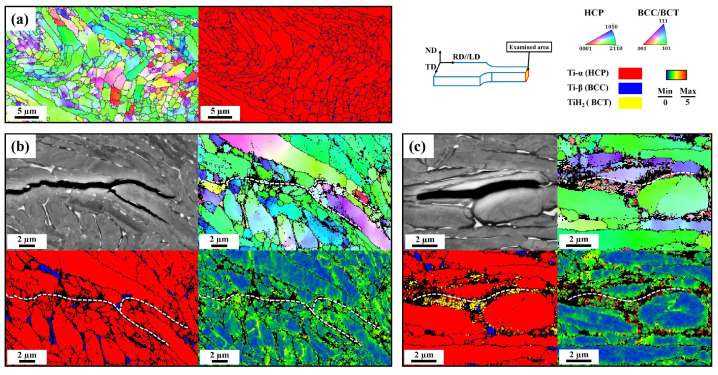
Deformed microstructures in the uncharged sample and hydrogen-assisted cracking behavior in the hydrogen-charged samples: (**a**) IPF and phase maps of the uncharged sample; ECCI and EBSD maps (IPF, phase map, and kernel average misorientation (KAM)) showing hydrogen-assisted crack propagation at the secondary crack on the cross-section (below ~1 mm from the fracture surface) in (**b**) the SH sample and (**c**) the HP sample.

Figure 5 shows deformed microstructural images in the uncharged sample (a) and the hydrogen-charged samples (b,c) depicting hydrogen-assisted cracking behavior, specifically focusing on the secondary cracks observed in the cross-section view (ND–TD). It is crucial to emphasize that only hydrogen-charged samples exhibit secondary cracks near the fracture surface, which is indicative of hydrogen-assisted cracking behaviors [36]. The uncharged sample displays a colony structure (Figure 5a), while an intergranular cracking was clearly observed in the SH sample, as the crack exclusively propagated along the grain boundaries as well as the α/β interface, supporting the notion that the nano/micro-voids generated at the GBs or the α/β interface could be a preferred site for HE. Indeed, GBs are reported as preferred pathways for the diffusion and accumulation of solute hydrogen [26,27,28,29]. Furthermore, the differences in hydrogen solubility and diffusion rates of α and β phases also contribute to the α/β interface serving as a hydrogen trap [7,11,18,25]. On the contrary, HP-assisted cracking is demonstrated in Figure 5c, showcasing crack propagation in zone III of the HP sample. Herein, the cracks predominantly advanced through the HP at the grain boundaries. As discussed in the preceding sections, the α phase-to-HP transformation results in local strain within the HP. This phenomenon significantly contributes to both the initiation of nano/micro-cracks and the promotion of crack propagation. Consequently, the HP plays a crucial role in the crack propagation process. Overall, EBSD analyses confirmed the distinct influences of SH and HP in intergranular hydrogen-assisted cracking.

## 4. Conclusions

This study investigated the susceptibility to hydrogen embrittlement of the Ti6Al4V alloy through a series of nanoindentation measurements, tensile tests, and microstructural characterization by comparison between hydrogen-charged and uncharged samples. The main conclusions of this work can be summarized as follows:

(1) Solute hydrogen and the hydride phase were successfully induced by different electrochemically hydrogen-charging processes conducted at low (1 mA/cm^2^) and high (50 mA/cm^2^) current densities, respectively;
(2) Hydrogen-charged samples generally exhibited brittle fracture features. On the microscopic scale, the impact of different hydrogen-charging conditions was not significant as evidenced by the relatively consistent results of nanoindentation measurements. However, macroscopic fracture behavior strongly depended on the diffused hydrogen concentration. With a low diffused hydrogen concentration indicating the presence of solute hydrogen, elongation was primarily reduced by 7.07% in the UE and 11.32% in the TE, respectively, but the strength was negligible. Conversely, when the hydrogen concentration was sufficient to form the hydride phase, a substantial reduction by 13.82% in the UE and 32.85% in the TE was found;
(3) Both solute hydrogen and the hydride phase give rise to hydrogen embrittlement. Solute hydrogen enhanced intergranular cracking behavior by forming nano/micro-voids at the grain boundaries. Conversely, the occurrence of cleavage or intergranular fracture modes depended on the distribution of the hydride phase. The increased volume within the hydride phase could act as sites for crack initiation and propagation, leading to more severe hydrogen embrittlement.

## Figures and Tables

**Table 1:** Mechanical properties of the hydrogen-charged (SH sample and HP sample) and the uncharged samples.

Mechanical Properties	Uncharged Sample	SH Sample	HP Sample
Ultimate tensile strength (MPa)	986 ± 6	1000 ± 6	963 ± 18
Yield strength (MPa)	904 ± 16	900 ± 13	874 ± 10
Uniform elongation (%)	7.07 ± 0.80	6.57 ± 0.05	6.27 ± 0.16
Total elongation (%)	13.24 ± 1.25	11.41 ± 1.53	8.89 ± 1.70

## Data Availability

The data that support the findings of this study are available from corresponding author upon reasonable request.
